# Insect neuropeptides as agents for pest control: potential and challenges

**DOI:** 10.1186/s40659-025-00651-0

**Published:** 2025-12-26

**Authors:** Natalia Konopińska, Szymon Chowański, Jan Lubawy, Paweł Marciniak, Karolina Walkowiak-Nowicka, Guy Smagghe, Arkadiusz Urbański

**Affiliations:** 1 Department of Animal Physiology and Developmental Biology, Faculty of Biology, Adam Mickiewicz University, Poznań, Poland; 2 Institute of Entomology, Guizhou University, Guiyang, 550025 China; 3 Department of Biology, Vrije Universiteit Brussel (VUB), 1050 Brussels, Belgium

**Keywords:** Integrated pest management, Neuroendocrine system, Peptidomimetics, RNAi, CRISPR

## Abstract

Chemical insecticides play a crucial role in securing global food production but have also caused serious environmental and health problems due to their persistence and low target specificity. In response, insect neuropeptides, which are biological macromolecules that act as key regulators of development, metabolism, reproduction, and behavior, are being explored as potential environmentally friendly alternatives for pest control. This review evaluates the prospects and limitations of using neuropeptides and their synthetic analogues as bioinsecticides. We discuss their mechanisms of action, challenges in increasing biostability, and the risks of affecting nontarget species. Strategies to improve, introduce and increase their applicability usage include peptide modification, targeted delivery systems, and the use of molecular techniques such as RNA interference (RNAi) and CRISPR-Cas9 to disrupt neuropeptide signaling pathways with high specificity. Advances in omics technologies and artificial intelligence are accelerating the discovery and design of novel neuropeptide-based agents. Nonetheless, regulatory challenges, high production costs, limited ecological impact data, and the potential for resistance development remain key obstacles. The integration of neuropeptide-based approaches with existing pest control methods, particularly within genetically modified crops and integrated pest management (IPM), could enhance both efficacy and environmental sustainability. Although the direct application of neuropeptides is still limited, targeting neuropeptide-related genes appears to be a promising and practical direction for the future of biological pest control.

## Introduction

One of the important inventions that has affected the rapid increase in the human population has been the use of large-scale chemical pesticides, which can meet the constantly growing food demand. Current estimations assume that total food demand will increase by approximately 35–56% between 2010 and 2050 [1, 2]. This has led to a significant rise in the use of pesticides, including insecticides, with over four million tons used annually on more than 60% of agricultural land. Although insecticides protect food production, they are also dangerous pollutants that reduce biodiversity and impact on beneficial animals such as pollinators. Moreover, they contaminate soil and water, may pass to the trophic level in different food chains, and pose health risks, causing approximately 200,000 deaths annually in developing countries [3]. For these reasons, better pest management strategies that maintain biodiversity, reduce the use of classical insecticides, and increase the safety of new methods are needed. These are global problems, and many governments see them. Therefore, for example, the EU directive 2009/128/EC aims to promote sustainable pesticide use and Integrated Pest Management (IPM). For many years, one of the groups of compounds considered as a new class of potent insecticides has been insect neuropeptides.

**Fig. 1:**
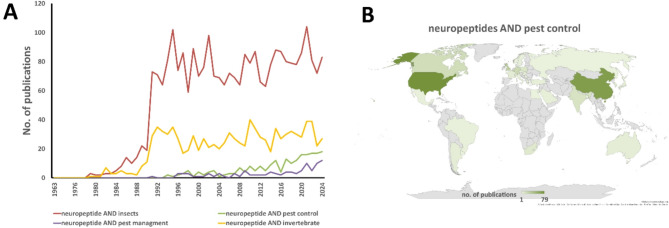
Temporal distribution of publications concerning insect neuropeptides and pest control/pest management (**A**). Figure B—Spatial analysis of countries especially involved in research concerning neuropeptides in pest control. The analysis is based on the number of publications related to these issues published by researchers from certain countries. The separation between “pest control” and “pest management” reflects the fact that different sets of articles are retrieved depending on the search term. The darker the shaded area is, the greater the number of publications. Data were retrieved from the Web of Science (https://www.webofscience.com, accessed 25.03–01.04.2025). The search terms are indicated on certain graphs. The analysis was performed via Microsoft Office 356 software (Adam Mickiewicz University licence)

Insect neuropeptides are among the most important groups of insect hormones that regulate a wide spectrum of physiological processes, starting with growth and development and ending with energy metabolism, food intake, and mating behavior [4–6]. Unsurprisingly, together with a better understanding of the role of neuropeptides in the regulation of insect physiology, an increasing number of articles concerning the use of neuropeptides in pest control have been published (Fig. 1). Moreover, we show that the number of publications concerning this issue will constantly increase. Furthermore, the expression of the idea of using neuropeptides in pest control was the nEUROSTRESSPEP project supported by the European Commission (Horizon 2020 Research and Innovation Program, 2015–2019), which assumed the development of selective control agents of insect pests on the basis of their peptide hormones and their synthetic mimetics [7]. Despite these examples and prognoses, we should ask ourselves whether we are closer to the introduction to the market of neuropeptide-based insecticides and whether it is possible to develop new pest management strategies on the basis of neuropeptide/neuropeptide signaling.

Unlike other articles concerning this issue, we do not focus strictly on the selection and description of neuropeptide families, which possess properties that are useful in controlling pest populations. The main emphasis was placed on critical analyses of limitations and challenges concerning the use of neuropeptides and their analogues as insecticides or as support for currently used plant protection products (PPPs), including their biostability, specificity, and legal issues related to their use. In addition to the development of molecular techniques, we discuss the potential use of techniques that allow manipulation of neuropeptide pathways, such as RNA interference (RNAi) or the CRISPR-Cas9 method.

## Direct usage of neuropeptides—is it possible?

### Insect neuropeptides—general information

**Fig. 2:**
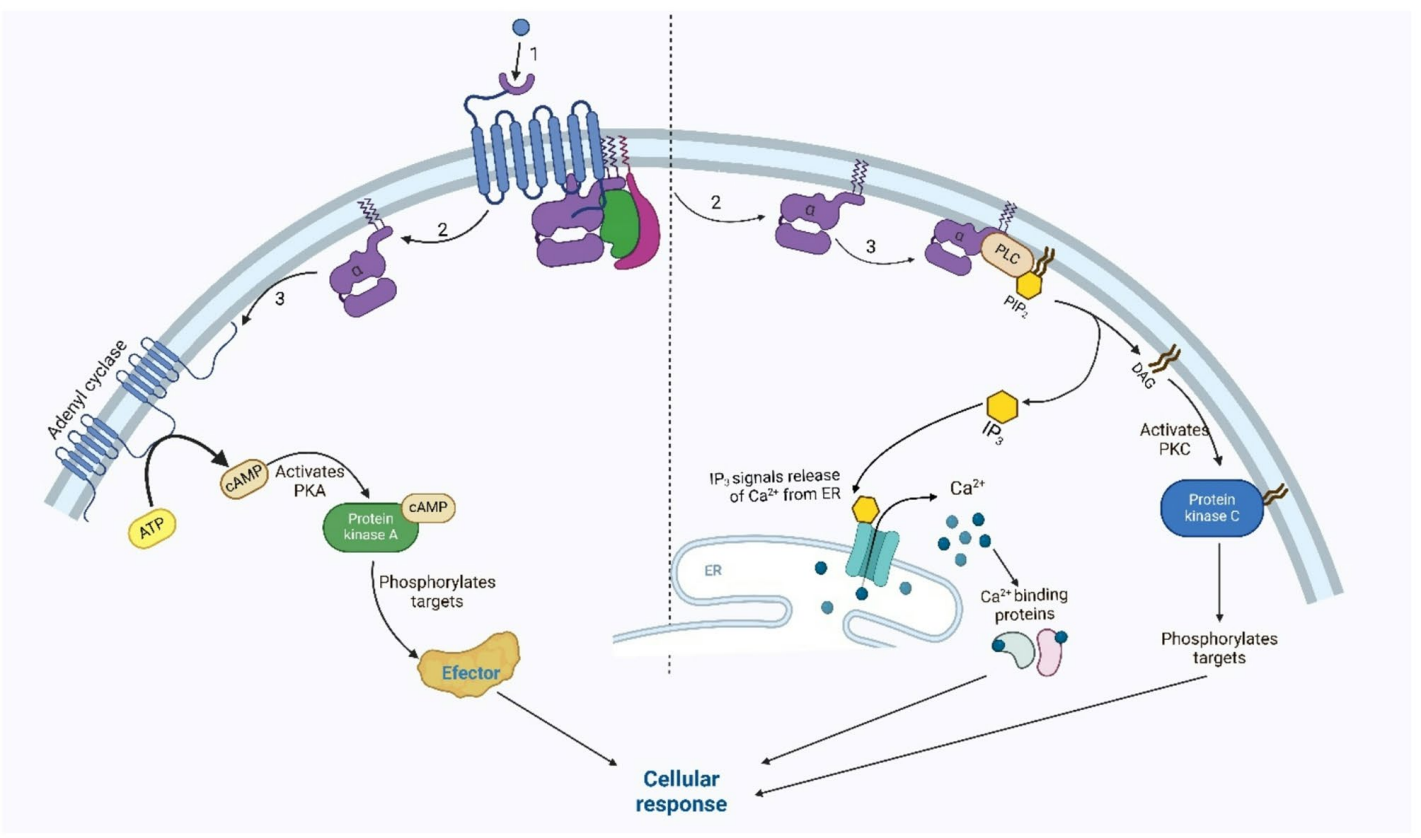
Schematic representation of insect neuropeptide activation of the signaling cascade in the cell. 1—Neuropeptide binding to a specific G-protein-coupled receptor, which leads to 2—dissociation of the α-subunit of the G-protein; 3—activation of either adenyl cyclase, which catalyzes the formation of cAMP, or phospholipase C, which hydrolyses membrane-bound phosphatidylinositol 4,5-bisphosphate (PIP_2_), resulting in the formation of the secondary messengers diacylglycerol (DAG) and inositol-1,4,5-triphosphate (IP_3_). cAMP activates protein kinase A (PKA), which phosphorylates effector proteins, while IP_3_ binds to calcium pumps on the endoplasmic reticulum (ER), releasing Ca^2+^, another second messenger, into the cytoplasm, which binds to a variety of proteins, activating a cascade of enzymatic pathways. The DAG formed from hydrolyzed PIP_2_ activates protein kinase C (PKC), which phosphorylates target proteins. All of these factors lead to a cellular response. The figure was prepared via BioRender software (https://www.biorender.com)

Neuropeptides are small, bioactive molecules that serve as key regulators of physiological and behavioral processes in insects. They function primarily as neurohormones, regulating major physiological processes such as growth, reproduction, metabolism, and stress responses. In addition, they can also act as neurotransmitters and neuromodulators. Insect neuropeptides exert their effects mostly by binding to G-protein-coupled receptors (GPCRs) on target cells (Fig. 2). Typically, the activation of GPCRs [8] is followed by the generation of soluble second messengers such as cyclic adenosine-3′,5′-monophosphate (cAMP) [9–11], inositol 1,4,5-trisphosphate (IP_3_) [1, 12], diacylglycerol (DAG) and calcium ions (Ca^2+^) [13].

Neuropeptides influence tissues directly or by modulating signaling pathways. Generally, the modes of action of different neuropeptides differ significantly. As an example of direct influence, we chose the well-known adipokinetic hormone (AKH) pathway, which regulates energy metabolism by mobilizing reserves in the fat body. When it is released from the *corpora cardiaca*, it binds to its receptor (AKHR). AKHR transduces the signal from the peptide to downstream effectors via two separate second-messenger systems [14, 15]. One activates adenylate cyclase, increasing cAMP, which activates protein kinase A (PKA) and phosphorylates downstream targets [16, 17]. The second pathway activates phospholipase C, producing IP_3_ from phosphatidylinositol 4,5-bisphosphate (PIP2), which increases Ca^2+^ via endoplasmic reticulum (ER)-resident IP_3_ receptors [for a review, see [18], leading to the breakdown of lipids and carbohydrates and providing energy for flight and other energy-intensive activities [19, 20]. Disruption of either the *AkhR* or *Akh* gene hinders triacylglycerol (TAG) mobilization in *Drosophila* [21–24]. The opposite effect on lipid metabolism is exerted by insulin-like peptides (ILPs) secreted from insulin-producing cells (IPCs), which bind to a single insulin receptor (InR). The binding of ILPs to InRs triggers the initial activation of phosphatidylinositol-3-phosphate kinase (PI3K) [25, 26], leading to the suppression of FOXO (Forkhead box subgroup O). Consequently, ILP signaling leads to the transcriptional repression of lipolysis [20].

On the other hand, insect neuropeptides can also exert indirect effects by modulating other signaling systems. For example, allatostatins (ASTs) and allatotropins (ATs), which possess diverse biological activities, regulate the production of juvenile hormone (JH, a sesquiterpenoid) in the *corpora allata*. JHs are among the most important insect hormones that regulate insect development and reproduction. ASTs inhibit JH biosynthesis, whereas ATs stimulate it [27–29].

Short neuropeptide F (sNPF) and pigment dispersing factor (PDF) are other examples, as they both regulate IPCs and their release of ILPs [30]. sNPF, which is released by lateral neurosecretory cells (LNCs), promotes ILP release and inhibits AKH secretion, thereby influencing carbohydrate homeostasis [30, 31]. Therefore, carbohydrate homeostasis is indirectly affected. Other neuropeptides, such as ATs and tachykinin-related peptides (TRPs), also interact with IPCs [32, 33]. However, these relationships between insect neuropeptides can be multiplied by the complexity of the hormonal regulation of homeostasis. These complex interactions highlight the challenges of using neuropeptides as insecticides, a topic explored later in this review.

### Selection of neuropeptide families depending on the goal

As mentioned in the previous section, neuropeptides play essential roles in insect physiology, regulating multiple aspects directly and indirectly. Unlike conventional pesticides, which often affect a broad spectrum of insects, neuropeptides offer the potential to develop highly specific insecticides that target particular physiological processes in selected species. On the basis of the literature data, we selected four areas that are especially important for controlling pest species populations. These include (1) feeding and digestion; (2) reproduction, mating and sexual behaviour (including pheromone synthesis) (3) locomotion and flight and (4) growth and development. Examples of neuropeptides important for the regulation of these physiological processes in selected pest species are summarized in Table 1.

**Table 1:** Examples of neuropeptide families that may be used in pest control can be divided into four areas: (1) feeding and digestion; (2) reproduction, mating and sexual behavior (including pheromone synthesis); (3) locomotion and flight and (4) growth and development

Area	Neuropeptide family	Example of action	Order	References
Feeding and digestion	Adipokinetic hormones	stimulate glycolysis in fat body and increase trehalose and LDL level in hemolymph of *Locusta migratoria*	Orthoptera	[32, 122–124]
		decrease level of stored carbohydrates of starved *Drosophila* flies	Diptera	[4, 125]
		promote the mobilization and breakdown of triacylglycerides in *Drosophila suzukii*	Diptera	[126]
	Allatostatins	increase protease and α-amylase release in gut of *Spodoptera littoralis*	Lepidoptera	[127]
	Allatotropins	increase frequency and amplitude of foregut contractility of *Helicoverpa armigera* and *Lacanobia oleracea* larvae	Lepidoptera	[128–131]
		suppress feeding of *Spodoptera frugiperda*	Lepidoptera	[5]
		inhibits amylase and trypsin release of *Spodoptera frugiperda*	Lepidoptera	[132]
	Corazonin	neurotransmitter of the Gr43a taste receptor in *Drosophila suzukii*	Diptera	[133]
	FaLPs (FMRFa-like peptides)	increase amount of secret from salivary gland of *Locusta migratoria*	Orthoptera	[129]
		stimulate salivary gland secretion in *Schistocerca gregaria*	Orthoptera	[128, 129, 134 , 135]
	Insulin-like peptides	activate glycolysis and inhibits gluconeogenesis in *Drosophila suzukii*	Diptera	[136]
		the knockdown of insulin-receptor genes increases the trehalose levels in the hemolymph of *Maruca vitrata*	Lepidoptera	[137]
	Myosuppressins	inhibit food intake in* Spodoptera littoralis*	Lepidoptera	[5]
	Neuropeptide F	induces continuous feeding in *Drosophila* larvae	Diptera	[5]
		increases food uptake *Schistocerca gregaria*	Orthoptera	[5]
		reduces glycogen and total lipid levels in fat body of *Helicoverpa armigera* and *Ostrinia furnacalis*	Lepidoptera	[5, 61, 62]
		regulate feeding and digestion of *Helicoverpa zea*	Lepidoptera	[138]
	Proctolin	increases midgut tension of *Locusta migratoria*	Orthoptera	[128, 139]
		stimulates foregut contractions of *Schistocerca gregaria*	Orthoptera	[128, 140, 141]
	Sulfakinins	stimulate the release of digestive enzyme (α-amylase) in *Rhynchophorus ferrugineus*	Coleoptera	[142]
		increase contractions of hindgut muscles of *Locusta migratoria*	Orthoptera	[4]
		reduce food intake in the desert locust *Schistocerca gregaria*	Orthoptera	[143, 144]
		stimulate food intake of *Tribolium castaneum* larvae	Coleoptera	[145]
		reduces food uptake and reduces digestive enzyme secretion from midgut and gastric caeca in *Locusta migratoria*	Orthoptera	[146]
		increase amylase release in Lepidoptera	Lepidoptera	[147, 148 ]
	Tachykinin-related peptides	regulate intestinal lipid production and control lipid balance in *Drosophila*	Diptera	[149]
		regulate starvation in *Hyphantria cunea*	Lepidoptera	[150]
		stimulate hindgut contractions of *Locusta migratoria*	Orthoptera	[128, 151, 161]
		regulate activity of hindgut of *Locusta migratoria*	Orthoptera	[151]
	Adipokinetic hormones	increase sensitivity for novel source of energy in *Spodoptera frugiperda*	Lepidoptera	[43]
	Neuropeptide F	control appetite memory in *Drosophila* flies	Diptera	[152,153]
		reduction appetite for food after NPF knockdown in *Acyrthosiphon pisum*	Hemiptera	[154]
	Short neuropeptide F	stimulate response to ethyl acetate and reduce antennal responses to odorants of *Bactrocera dorsalis*	Diptera	[5, 155, 156]
		knockdown of *snpf* gesne influences the feeding behavior of* Acyrthosiphon pisum*	Hemiptera	[157]
		sNPF knockdown significantly decreased food intake in* Rhopalosiphum padi*	Hemiptera	[158]
		increase for food-seeking behavior of *Leptinotarsa decemlineata*	Coleoptera	[159]
Reproduction and mating (including pheromone synthesis)	FaLPs (FMRFa-like peptides)	increase contraction frequency of ejaculatory duct, transparent accessory gland, and seminal vesicle muscles of *Tenebrio molitor*	Coleoptera	[160, 161]
		increase in ovaries and oviducts muscle tone of *Tenebrio molitor*	Coleoptera	[160, 162]
	Insulin-like peptides	increase number of dividing cells in ovarioles and stimulate vitellogenesis of *Maruca vitrata*	Lepidoptera	[137, 163]
		regulate spermatogenesis in *Drosophila*	Diptera	[163, 164]
	Natalisin	*Natalisin-RNAi* defects in the mating behaviors in *Drosophila*	Diptera	[165]
		*Natalisin-RNAi* reduces the fecundity in *Tribolium castaneum*	Coleoptera	[165]
		*Natalisin-RNAi* influences sexual behavior and the mating rate in *Spodoptera*	Lepidoptera	[43]
	Neuropeptide F	controls ovarian maturation of *Schistocerca gregaria*	Orthoptera	[166]
		controls courtship behaviors of *Schistocerca gregaria*	Orthoptera	[167]
	Short neuropeptide F	decreases the total sperm number of *Tenebrio molitor*	Coleoptera	[160]
		controls contractions of ejaculatory duct of *Tenebrio molitor*	Coleoptera	[160]
		stimulate egg development in *Locusta migratoria*	Orthoptera	[166]
		sNPF silencing reduced the number of nymphs per female of *Acyrthosiphon pisum*	Hemiptera	[157]
		decrease oviduct muscle contraction and number of laid eggs in *Tenebrio molitor*	Coleoptera	[160]
	PBAN	regulates sex pheromone biosynthesis in *Spodoptera litura*	Lepidoptera	[99]
		regulates synthesis of sex pheromone biosynthesis in female *Heliothis peltigera*	Lepidoptera	[4]
		regulates fecundity of* Spodoptera frugiperda*	Lepidoptera	[168]
		stimulate production of sex pheromones in *Plutella xylostella*	Lepidoptera	[169]
	Adipokinetic hormones	modulates the sexual behavior and flight duration of males and fecundity of females of *Bactrocera dorsalis*	Diptera	[170]
	Tachykinin-related peptides	stimulate contractions of muscle in oviducts of locust	Orthoptera	[171–173]
		regulate fecundity in *Tribolium castaneum*	Coleoptera	[165]
	Sulfakinins	stimulates food-seeking behavior while reducing mating activity of* Bactrocera dorsalis*	Diptera	[109]
Locomotion and flight	Adipokinetic hormones	during initial phase of flight involved in release of trehalose to the hemolymph in *Locusta*	Orthoptera	[174, 175]
		in stable flight it activates triacylglycerol lipase in *Locusta*	Orthoptera	[174, 175]
	CCH-amide	decreased expression of genes encoding CCH-amide causes reduced locomotory activity of *Drosophila*	Diptera	[5]
	Leukokinins	involved in regulation of sleep and locomotory activity of *Drosophila*	Diptera	[5, 176]
Growth and development	Neuropeptide F	affects the development and growth of *Plutella xylostella* larvae	Lepidoptera	[177]
	Insulin-like peptides	growth deficiency in *Drosophila* with reduced insulin signaling	Diptera	[178]
	Allatostatins	prolongation of the larval phase by stimulating JH synthesis in *Spodoptera frugiperda*	Lepidoptera	[179]
	Allatotropins	inhibit JH biosynthesis, accelerating metamorphosis processes in *Spodoptera frugiperda*	Lepidoptera	[179]

### Potential use of neuropeptides and methods of their delivery

Despite the broad physiological action of insect neuropeptides, there are still no available insecticides based on them. This may be because these hormones are peptides, and their use as potential bioinsecticides in the field faces several important obstacles associated with both their evolutionary conservation and practical aspects of their application. The first important question is neuropeptide specificity and their possible action on nontarget species. No less important issue in the consideration of neuropeptides as insecticides is the fact that peptides are fragile to environmental factors such as low/high temperature, UV radiation, low/high pH, and humidity, which can be important for their stability. Moreover, they can be degraded by proteolytic enzymes [34–37]. This is why major research currently and in the future should focus on overcoming those obstacles. This means that one should look for a way to increase the biostability and specificity of peptides or for a way to deliver neuropeptides to target insects with unchanged bioactivity.

#### Specificity

One major issue with current insecticides is their role as environmental pollutants due to their low target specificity, which harms nontarget species and reduces biodiversity. In particular, their effects on beneficial organisms such as pollinators and detritivores are concerning. Therefore, a key challenge in developing eco-friendly pest control methods is minimizing off-target risks.

Although insect neuropeptides naturally regulate many physiological processes, their external application could also affect nontarget species. The structural similarities between insect neuropeptides and those in other arthropods raise concerns about cross-reactivity, making unmodified neuropeptides a less viable option. However, their distribution is not uniform across insect groups. For example, while natalisins (NTLs) and kinins are widespread, their functions vary [38–40]. Honeybees (*Apis mellifera*) lack both NTL and its receptor, unlike pests such as *Varroa destructor*, which possess a functional NTL system, suggesting selective control strategies with minimal risk to honeybees. Conversely, kinin signaling is absent in Coleopteran pests, and proctolin is missing in Lepidopterans such as *Spodoptera frugiperda*, limiting their use [41–43]. Thus, developing neuropeptide-based pest control strategies requires careful consideration of these differences to prevent unintended ecological consequences.

#### How to improve neuropeptide biostability—agonists and antagonists

In situations when we want to activate the receptors, we need to design an agonist that is biostable and has a long biological half-life but still has high efficiency in interacting with the receptors. Here, we need to cope with susceptibility to degradation, poor bioavailability and solubility, or rapid clearance of neuropeptides. This can be solved in two ways: by modifying the native neuropeptide to increase its biostability or by searching for nonpeptide molecules that mimic neuropeptide functions as agonists.

To obtain a compound with specific properties by modifying the structure of the native peptide, first, the core sequence responsible for binding to the receptor must be determined. There is also a need for good characterization of the ligand binding domain in a receptor. In the past, many time-consuming ligand-receptor docking (structure–activity relationship (SAR)) studies were performed. Today, computational tools provide powerful new opportunities to predict receptor structures and analyze structure–activity relationships much faster and more efficiently. AlphaFold is an example of such powerful tool, which enables high-accuracy protein structure prediction from sequence alone. It makes it especially valuable in SAR studies by allowing structure-based design even for targets lacking experimental data. Using such techniques allows for hypothesis generation and supports peptide engineering, particularly in non-model organisms [44].

The modifications used in the synthesis of peptidomimetics include the substitution of a single or several amino acids and the introduction of nonprotein amino acids or their non-amino acid analogues. Moreover, additional chemical compounds with extended side chains are introduced, which promote the creation of a steric barrier and thus provide protection of the peptide bond against the action of hydrolytic enzymes [45, 46]. An example can be incorporation into the peptide chain of ω-unsaturated amino acids or β-substituted Cys derivatives. Another appealing and broadly used technique lies in backbone amide replacement with amide bonds that look like surrogates or isosteres. Isosteric replacement modifies the backbone and therefore inhibits protease activity at the substrate cleavage site [45]. Moreover, cyclization is an important and attractive way to increase biostability [47]. These include end-to-end, side-chain-to-side-chain, and side-chain-to-end cyclization coupled with back-side chain modification. The method used for cyclization is backbone cyclization through the connection of the *N* ^α^ and/or *C* ^α^ atoms in the peptide backbone to each other or to side chains or to the carboxyl and amino ends with ω-amino, ω-carboxy, and ω-thioalkyl amino acids [47–49].

The opposite approach involves the search for antagonists that inhibit neuropeptide receptors. This can be accomplished in two ways: the use of neuropeptide-based antagonists (competitive antagonists) or the use of nonpeptide antagonists (inhibits binding and/or signal transduction via allosteric effects) [50]. First, we need to address similar problems, such as those associated with peptidomimetic synthesis. The second solution has one major advantage: it does not consider problems concerning the stability of peptides. On the other hand, in that way, the high selectivity of the inhibition of specific receptors can be lost. The goal is to find an antagonist molecule with high affinity for the receptor but low efficiency in activating it. Examples of different modifications used to increase the biostability of agonists or antagonists of neuropeptides considered insecticides are presented in Table 2.

**Table 2:** Examples of different derivatives and modifications used to increase the biostability of neuropeptides

Type of modification	Example of modifying compound	Location in the chain			Example of neuropeptide	Reference
		*N-*terminus	Inside chain	*C*-terminus		
Substitution of amino acids	α-methyl-Phe	x	x		kinins	[180]
	β^3^Phe	x	x			[180]
						[181]
	β^2^Trp	x	x			[182]
	β^2^homoPhe	x	x			
	β^3^Pro	x	x			
	β^3^Trp	x	x			
	*O*-t-butyl-L-hydroxy-Pro	x				[182]
	t-butyloxycarbonyl-L-Trp	x				
	L-aspartic acid beta-t-butyles	x			myosuppressin	[182]
	cyclopropyl-Met		x			
	*O*-methyl-Tyr	x			proctolin	
	*O*-ethyl-Tyr		x			
	*O*-propargyl-Tyr		x			
	*p*-fluoro-Phe		x			
	*p*-chloro-Phe		x			
	*p*-bromo-Phe		x			
	*p*-amino-Phe		x			
	*p*-nitro-Phe		x			
	*p*-azido-Phe		x			
	*O*-methyl-Gly			x	myokinins	
	*O-*ethyl-Gly			x		
	*O*-phenylomethyl-Gly			x		
	*S*-methyl-Gly			x		
	4-phenylbutylamino-trans-DL-1,2-cyclopentanediacyl-Arg	x			PBAN	
	4-phenylbutylamino-trans-DL-1,2-cyclohexane-diacyl-Arg	x				
	4-phenylbutylamino-cis-1,2-cyclohexane-diacyl-Arg	x				
	9-phenyl-nonyl-Arg	x				
	D-Phe		x			
	hydrocinnamyl-Ala		x		allatostatins	[183]
	cyclopropyl-Ala		x			
	2-amino-2-indancarboxylic acid, beta-cyclopropyl-L-Ala		x			[184, 185]
	hydrocinnamyl-L-Ala	x				[48]
by nonpeptide amino acids	α-aminoisobutyric acid (Aib)	x	x	x	kinins	[186]
						[187]
						[100]
						[180]
						[188]
	multi-Aib	x	x	x		[180]
						[188]
						[189]
						[187]
						[100]
	aminohexanoic acid (Ahx)	x				[188]
	2-amino-2-indancarboxylic acid		x		allatostatins	[184, 185]
by nonamino acids compounds	Ψtetrazole and different 1,5-disubstituted tetrazole ring		x		kinins	[188]
						[188]
						[49]
	succinic acid		x		myosuppressins	[190]
	4-(1H-benzo[d][1,2,3]triazol-1-yl)-4-oxobutanoic acid		x		allatostatins	[183]
	minoindane carboxylic acid (Aic)		x			
	L-1,2,3,4-tetrahydroisoquino line-3-carboxylic acid (Tic)		x			
	(E)-3-(4-nitrophenyl) acrylic acid		x			[191]
*N*-end modification	cinnamic acid	x			kinins	[186]
	*p*-nitrocinnamic acid	x				
	*p*-methylcinnamic acid	x				
	acetic acid	x				
	pivalic acid	x				
	2-hydroxyisobutyrylic acid	x				
	acrylic acid	x				
	2-methylacrylic acid	x				
	hydrocinnamic acid	x			PBAN	[192]
	9-fluoreneacetic acid	x			FTPRLamide	[193]
					PBAN	[192]
	6-phenylhexanoic acid	x			FTPRLamide	[193]
	1-pyrenebutyric acid	x			PBAN	[192]
	7-bromofluorenetic acid	x				
	succinic acid	x			myosuppressin	
	sebacic acid	x				
	B_10_ Boran cluster of butanoic acid	x			myokinins	
backbone cyclization of peptides	cycloproctolin				kinins	[188]
					PBAN	[50]
					proctolin	[192]
nonpeptide agonists/antagonists	CP-96345-1				tachykinins	[192]
	benzethonium chloride				myosuppressin	

Theoretically, each receptor for each neuropeptide can be considered as a target in pest management through the use of an agonist or antagonist system. Nevertheless, it seems that only receptors for insect-specific neuropeptides have a chance to be a star in that field. Thus, for example, insect ILPs seem to be less useful in the search for insecticides. This is because some insect neuropeptides can act on receptors of different species, including nontarget individuals. Thus, the possible cross-reactivity between neuropeptide receptors from different animal groups needs to be considered. Considering the activation mechanism of GPCRs, in the search for new insecticides, agonists and antagonists as activators and inhibitors/blockers can be used to modify specific pathways that can be activated or inhibited. The combination of allosteric modulators with orthosteric ligands can also be used to increase/maintain the specificity of insecticides. Thus, the high diversity of neurohormone GPCRs provides many candidates with strong species-specific properties with low off-target probabilities. Hence, it is still quite easy to find a good candidate but difficult to reach the final step as a new insecticide on the market [51]. A list of potential candidates of native neuropeptides and their analogues is available, for example, on the FreePatentsOnline.com website.

While insect neuropeptide analogues are designed to be highly specific to target pests, there is a risk that insects could develop resistance over time, similar to what has been observed with traditional chemical insecticides [52]. Additionally, the synthesis of these analogues can be complex and costly. Producing them in large quantities for widespread agricultural use may present significant economic and technical challenges. Another possible disadvantage of using neuropeptide mimetics is the lack of sufficient knowledge on their long-lasting effects on ecosystems. Although these analogues are generally considered environmentally friendly, their long-term effects on nontarget organisms and ecosystems are not fully understood [53]. All of the above mentioned factors highlight the importance of continued research and careful consideration in the development and deployment of insect neuropeptide analogues as pest control agents and careful consideration of all the advantages and drawbacks.

#### Methods of delivery

As we described previously, several systems have been developed to protect neuropeptides from environmental factors. These systems can be used either by topical application (apart from protection from environmental factors, which also increase the penetration of the insect cuticle) or by oral delivery via food. The second system also needs to protect neuropeptides from the extremely low pH of the insect alimentary tract and enhance the penetration of the peritrophic membrane. A summary of potential methods is presented in the Table below (Table 3).

**Table 3:** Summary of predictions of potential methods of neuropeptide delivery [35, 36, 61, 62, 194, 195]

Possible way of delivery			
	Advantage	Disadvantage	Possibility
Topical application	- ease of application - lack of degradation by digestive enzymes - low cost of application	- low efficiency of penetration through hydrophobic cuticle - limited molecular size - low selectivity—affects all insects presented on field - the need to increase hydrophobicity - possible toxicity of carrier solvent (usage of hydrophobic solvents) - sensitivity to sunlight - weather-dependent efficiency	- modified peptides with enhanced biostability - liposomes - nanocarriers
Delivery with food	- ease of application - selectivity to herbivores - low limitation of molecular size - lower cost of application	- degradation by digestive enzymes - degradation by gut microbiota - necessity of chemical modification to decrease sensitivity to hydrolysis - sensitivity to pH of gut lumen - weather-dependent efficiency - taste-dependent repellent activity	- modified peptides with enhanced biostability - liposomes - nanocarriers - transgenic organisms (bacteria, plants)
Type of modification			
Nanocarriers	- enhance penetration through cuticle - protection of neuropeptide from digestive enzymes and lower pH in insect gut	- toxicity of nanoparticles - low knowledge about the environmental long-term impact of nanoparticles - limited molecular size - high costs of production	
Liposomes	- enhance penetration through cuticle - protection of neuropeptide from digestive enzymes and lower pH in insect gut - protection from temperature and UV radiation	- technical problems with production - instability under field condition - limited molecular size - high costs of production	
Transgenic plants	- high selectivity to herbivores and to species feeding on a specific plant - low costs of usage - no need to make a field spraying	- possible allergic effects (consumption of plants by human) - high costs of plant modification - problems result from law and public acceptance of GMO	

## Modulation of the neuropeptide pathways

Despite extensive research, the practical use of neuropeptides and their analogues in pest control is limited and associated with many obstacles. However, their essential roles in insect reproduction, metabolism, feeding, and behavior have been well established. Advances in understanding neuropeptide function have enabled the use of RNAi and CRISPR for loss-of-function studies. This knowledge supports the development of alternative strategies targeting neuropeptide signaling pathways, such as the silencing of genes encoding neuropeptides, their receptors, or downstream components.

### RNA interference

#### RNAi in pest control

By enabling sequence-specific posttranscriptional gene silencing, RNAi has become a powerful tool in functional genomics, pest control, and insect physiology. This approach allows for the targeted disruption of gene expression, offering valuable insights into gene function and supporting the development of innovative pest management strategies. To understand the RNAi mechanism in detail, we recommend the book “RNA interference in Agriculture: Basic Science to Applications [54].

The initial research on the application of RNAi technology for pest control focused on laboratory experiments in which dsRNA was administered to insects via injection [55]. RNAi has been tested on pests such as beetles, butterflies, and true bugs. Injecting dsRNA targeting essential genes inhibited larval growth and development, and decreased survival [56, 57]. Researchers then shifted to more practical delivery methods, such as spraying plants or feeding insects dsRNA-containing plants. For example, in *Ostrinia furnacalis*, spraying or egg soaking with dsRNA targeting digestive enzymes (such as LIM protein 1 and chymotrypsin-like serine proteinase C3) led to 73–100% mortality. Advances in the understanding of RNAi have enabled its use in agriculture. A milestone was the development of transgenic maize by Baum et al. (2007), producing dsRNA targeting V-ATPase A, which increased larval mortality and reduced root damage from *Diabrotica virgifera*. Undoubtedly, all these studies contributed to the introduction of RNAi-based products to the market. First, SmartStax® PRO maize, approved by the EPA in 2017, expresses dsRNA targeting *Snf7*, which is essential for protein transport in *D. virgifera*, causing high larval mortality and effective crop protection [59]. The second product, Calantha® for the selective control of *Leptinotarsa decemlineata* (active ingredient Ledprona, which contains dsRNA targeting Proteasome Subunit Beta Type-5), was introduced in 2023/2024 and represents the first foliar-applied, sprayable dsRNA-based insecticide. This product is based on Spray-Induced Gene Silencing (SIGS), an approach in which dsRNA is applied directly onto plant surfaces, where it is absorbed and processed by the plant’s RNAi machinery to target specific pest genes. SIGS offers several advantages: it is environmentally friendly, reduces off-target effects on non-pest organisms, and can be applied to a wide range of crops. The approval of Ledprona by the EPA in late 2023 thus marks a significant milestone in the commercialization of SIGS technology [60].

In the case of insect neuropeptides, the use of the RNAi technique is generally limited to determining the mode of action of selected neuropeptides. However, the light at the end of the tunnel is research by Yue et al. [61, 62] on dsRNA targeted *NPF*, a gene for neuropeptide precursor which is considered as a feeding behavior regulator. Silencing *NPF* in *O. furnacalis* and *Helicoverpa armigera* led to lowered food intake, reduced body mass, and metabolic disruption. However, most importantly, transgenic maize, tobacco, and cotton expressing dsRNA against *NPF* reduced larval feeding pressure [61, 62].

#### Challenges and limitations in the use of RNAi control of pest species related to neuropeptides

As stated above, one of the main obstacles related to the use of neuropeptides in pest control is their low species-specificity. Considering that perspective, RNAi offers greater potential than the use of neuropeptides or peptidomimetics. Carefully designed dsRNA molecules can minimize effects on nontarget species. However, off-target effects can still occur if siRNAs partially match other genes. For example, dsRNA targeting *V-ATPase* genes in *D. virgifera* also reduced survival in *D. undecimpunctata* and *L. decemlineata* [58]. Studies also show that beneficial insects like lady beetles can also be affected. *Adalia bipunctata* and *Coccinella septempunctata* are not only sensitive to dsRNAs targeting their own genes, but they also respond to dsRNA targeting the V-ATPase A of the western corn rootworm, *D. virgifera virgifera* (Dvv dsRNA). However, the degree of susceptibility depends on how similar the dsRNA sequence is to their own genes. [63]. There is also concern that dsRNA or plant-derived microRNAs may be transferred through food chains. For example, rice-derived MIR168a was detected in serum and tissues of animals fed with transgenic plant, where it altered LDLRAP1 (low-density lipoprotein receptor adaptor protein 1) expression and cholesterol metabolism in mice [64].

Another significant obstacle in RNAi application is the variability of responses among insect species, mainly due to differences in digestive physiology, dsRNA uptake, and intracellular processing. Beetles (Coleoptera) typically exhibit strong RNAi responses, whereas Lepidoptera exhibit inconsistent and often weak effects [58, 65, 66]. This is exemplified by *Ostrinia nubilalis*, which is RNAi sensitive, in contrast to the resistant *O. furnacalis* [67]. Additionally, larvae are generally more responsive to dsRNA due to their smaller size and weaker defense systems [56, 66, 68].

Importantly, RNAi efficiency is influenced by digestive enzymes such as DNases and RNases, whose levels vary by species and diet [69]. Acidic midguts in beetles preserve dsRNA, whereas alkaline environments in Lepidoptera promote degradation [70, 71]. For this reason, oral and topical applications are less reliable than direct injection of dsRNA. The solution to this obstacle can be the improvement of more effective delivery methods, such as coating with polymers, lipids and nanocarriers, as well as the use of chitosan and liposomes [72]. These solutions also improve dsRNA stability and protect against environmental factors such as UV light, temperature, and microbial activity [72, 73].

Another limitation is the lack of RNAi amplification mechanisms in insects, which require repeated or continuous dsRNA administration, which is especially difficult under field conditions. Transgenic plants expressing insect-specific dsRNAs represent a promising solution. The oral ingestion of such plants by *D. virgifera* larvae has resulted in effective gene knockdown [59]. However, as with other control methods, resistance can develop. Mechanisms of resistance include mutations in target genes and reduced dsRNA uptake. In laboratory studies, resistance in *D. virgifera* developed within 11 generations when larvae were reared on transgenic maize expressing *DvSnf7* dsRNA [74]. This resistance was recessive and linked to a single autosomal locus and, importantly, was not limited to *DvSnf7* but affected other dsRNAs as well. The primary mechanism was reduced uptake by intestinal cells, which prevented effective silencing. These findings highlight the need for gene target rotation and the integration of RNAi into broader pest management strategies [75].

### CRISPR-Cas

The CRISPR-Cas9 system is a revolutionary method for gene editing that uses a guide RNA (gRNA) to target the desired DNA sequence and the Cas9 enzyme to cut the DNA at that location [76]. This break allows for the subsequent modification of the DNA. There are numerous excellent reviews on the precise mechanisms of this technique, which we encourage the reader to visit [77–79]. By enabling precise gene knockouts, knock-ins, and modifications, CRISPR–Cas9 allows researchers to investigate the functional roles of specific neuropeptides and their signaling pathways. For example, CRISPR-Cas9 has been used to disrupt genes encoding neuropeptides such as ILPs and PDFs in *Drosophila, S. frugiperda* or *Ae. aegypti*, revealing their roles in growth, metabolism, molting and circadian rhythms [80–82]. Future applications could include editing neuropeptide receptors to study their ligand specificity or developing pest-specific gene drives to disrupt neuropeptide signaling in target species.

The sterile insect technique (SIT) is a pest control method that involves the release of sterile males to reduce wild populations. Although, based on current literature, neuropeptides have not yet been widely implemented in SIT programs, ones that regulate reproduction, such as ASTs and ATs, or mating, such as PBAN, could be targeted to increase the effectiveness of SIT. For example, CRISPR-Cas9 could be used to disrupt neuropeptide genes essential for mating or fertility, creating sterile males or females. Ashok et al. [83] reported that disruption of the PBAN gene leads to mating disruption, especially in females. Mutant females are significantly less attractive to wild males, and crosses with wild males result in no fecundity [83], which is a promising alley for SIT [84].

#### Challenges and limitations of the use of CRISPR-Cas9 in pest control related to insect neuropeptides

However, the use of gene-editing technologies such as CRISPR-Cas9 and genetic control methods such as SIT raises some ethical and regulatory concerns. CRISPR-Cas9 holds promise for insect pest control, yet it faces several challenges and limitations. Off-target effects can lead to unintended mutations in nontarget genes, posing risks of unpredictable consequences [85]. Targeting neuropeptides with CRISPR-Cas9 for pest control could pose potential risks. Neuropeptides act through intricate networks of relationships with other neuropeptides and molecules to produce final phenotypic outcomes. Disrupting “one brick” could cause a domino effect, leading to unintended consequences or ineffectiveness of the whole procedure (e.g., lethal phenotypes, not suitable for use in SIT). Although neurohormone knockout trials are being conducted, knockout of JH acid methyltransferase (*JHAMT*) in *Ae. aegypti* induces mortality at the onset of metamorphosis [86], whereas knockout of gene encoding prothoracicotropic hormone (*PTTH*) in *S. frugiperda* causes extended larval development and death [82]. However, similar approaches targeting neuropeptides do not always result in lethality. For example, disruption of one of the neuropeptide hormones, PDF signaling affects reproductive capacity without causing lethality, demonstrating that neuropeptide manipulation can yield significant phenotypes beyond survival [87]. Second, efficient delivery methods are needed to fully utilize this method since the methods currently used are labor intensive and impractical for large-scale applications. At present, the predominant method for gene editing in pest insects involves the microinjection of Cas9 and gRNA components directly into insects [88]. Additionally, insect populations may develop resistance to CRISPR-Cas9-based interventions, akin to the resistance observed with insecticides. For example, CRISPR-based SIT, termed precision-guided SIT (pgSIT), was developed for mosquitoes and *Drosophila* [88, 89]. In greenhouse experiments, mutant strains created via these technologies have demonstrated high efficiency in suppressing populations. However, mutations at target sites can lead to allele resistance, which may reduce or eliminate the effectiveness of population suppression. Moreover, ethical and ecological concerns arise from the release of genetically modified insects into the environment, potentially affecting nontarget species [90, 91]. Furthermore, regulatory hurdles impose strict scrutiny, slowing research and implementation [91]. Despite these challenges, ongoing research aims to increase the precision, efficiency, and safety of CRISPR-Cas9 for effective pest management. It is crucial now to focus on improving CRISPR-Cas9-based pest management technologies and combining them safely with SIT, as this integration offers substantial potential for effective pest control [91]. Ensuring the safe and responsible use of these technologies will require collaboration among scientists, policymakers, and the public [92].

## Other aspects and research direction

### Exploring diversity and the use of artificial intelligence

In the past ten years, the rapid development of omics technology has introduced research on insect neuropeptides to the era of so-called big data. The use of genomics, transcriptomics, peptidomics, and metabolomics has generated large amounts of data. This opens new directions for research in this field. This can be divided into two major directions: (i) the discovery of new neuropeptides with unknown sequences, and (ii) the application of omics technology as well as other newly developed techniques in functional studies of neuropeptides, especially in lesser-known insect species. These findings may lead to the discovery of new neuropeptide-based insecticides.

(i) Discovery of new neuropeptides with unknown sequences.

The discovery of new neuropeptides not only in insects but also in other organisms can be challenging for several reasons [93]:

1. Neuropeptides occur in organisms at very low concentrations, sometimes up to 1000-fold or even lower than those of classical neurotransmitters and other metabolites. These concentrations also vary between tissues or body regions.
2. Neuropeptides are products of proteolytic processing of larger precursor proteins and posttranslational modifications (PTMs) that occur inside cells or during transportation.
3. Neuropeptides are prone to rapid degradation. Thus, it is often difficult to identify peptides as endogenous and not simply as the product of a degraded larger protein.
4. There is a large amount of variability between different neuropeptides, either owing to possession of different sequences but with the same mass or because they have different PTMs.
5. Neuropeptides can have the same structure but different functions or have different functions depending on the cell type and nearby receptors.
6. Isoforms of various neuropeptide families often exist, and the localization of specific neuropeptides can be challenging owing to difficulties in assigning mass spectral peaks to specific peptides.

Currently, mass spectrometry is the major method of choice for sequencing and determining the PTMs of neuropeptides [93, 94]. For several years, the term neuropeptidomics has been introduced for the global measurement and identification of the complement of peptides in a cell, tissue or extract from the brain or nervous system tissue. In terms of insects, it embraces the set of neuropeptides from a single species [95, 96]. Tremendous progress has been made recently in neuropeptide identification when peptidomics has been assisted [42, 97, 98]. For the last few years, relatively easy access to next-generation sequencing (NGS) has produced a large amount of data generated via only in silico analysis without sequence confirmation at the protein/peptide level [41, 99]. This information can be used to develop strategies in which the neuropeptide family of potential new bioinsecticides should be designed. For example, kinin neuropeptides have been shown to be perfect candidates for peptide-based biopesticides against aphids and are potentially safe for beneficial insects such as *Chrysoperla carnea,* a natural aphid predator, which has been shown to lack kinin signaling [100].

This has been supported by the creation of databases that include neuropeptides. For insect neuropeptides, the primary database currently available is DINeR [101], which was developed as a result of the previously mentioned nEUROSTRESSPEP project (https://neurostresspep.eu/). Other neuropeptide databases are also available, including those that focus on non-insect neuropeptides [102–105]. Moreover, several computational methods have been developed to identify neuropeptides or neuropeptide precursors. Most of them are based on machine learning algorithms. The most common methods used to be NeuroPID, NeuroPP and NeuroPRED [105, 106]. Some of them, such as NeuroPID or NeuroPRED, are no longer valid or have been updated [107]. Among them, a tool dedicated to insect neuropeptides is called NeuroPIpred [108]. The authors claim that their tool can discriminate between insect neuropeptides and non-neuropeptides with high accuracy and allow users to generate mutant analogues of neuropeptides, which can be potential neuropeptide-based insecticides. According to the website of the model, the newest dataset at the date of writing this review contains 2024 insect neuropeptides obtained from the DINeR database (https://webs.iiitd.edu.in/raghava/neuropipred/index.html). On the basis of all these methods for the prediction of neuropeptides from proteomes, the prediction of cleavage sites in neuropeptide precursors can be performed, or even whole neuropeptide precursors can be predicted. The younger model, NeuroPpred-SVM, predicts neuropeptides on the basis of the embeddings of bidirectional encoder representations from transformers and other sequential features via a support vector machine (SV) [106]. Compared with other available models (NeuroPIpred, PredNeuroP, NeuroPpred-Fuse, and NeuroPpred-FRL), this model had the lowest false positive rate, indicating high specificity and accuracy [109]. For example, SVM machine learning models have been successfully used to design allatostatin analogues with potential for use in pest management strategies [110]. All artificial intelligence (AI) methods, including machine learning, have the potential to significantly advance the search for new molecules that can be used as bioinsecticides. However, the use of these tools has several limitations that can lead to overinterpretation of the results. These mainly include the quality and quantity of the input data. If the data are incomplete or biased, this can affect the reliability of predictions. Additionally, owing to structural similarities among neuropeptides, the model might predict non-neuropeptide sequences as neuropeptides, leading to false positive results. Therefore, computational predictions require experimental validation to confirm the presence or functionality of predicted neuropeptides or neuropeptide analogues.

(ii) Functional studies of neuropeptides, especially in non-model insect species.

A better understanding of the physiological functions of neuropeptides is crucial for the development/design of novel bioinsecticides. Understanding neuropeptides functions is extremely difficult, as their effects can be tissue-specific or species-specific [111]. Furthermore, even slightly different neuropeptide isoforms from the same family can have different effects [112]. Very often, neuropeptides exert pleiotropic effects, affecting different processes. Additionally, a better understanding of the neuroendocrinological regulation of basic life processes in insects provides a broader perspective on the impact of potential neuropeptide-based pest control on nontarget individuals. Focusing on non-model insect species is particularly important to identify unique neuropeptide signaling pathways that could serve as selective targets for pest control. For example, in *O. furnacalis* knockdown of the neuropeptide F gene alters feeding behavior. Novel methods are needed to uncover the physiological roles of neuropeptides. These include injections of synthetic neuropeptides, dsRNA, or CRISPR-Cas9 gene editing. Each technique provides insights at different levels of organismal organization and can complement other approaches [113]. To match the local release kinetics and concentrations of neuropeptides with the required sensitivity and specificity of detection and at the same time provide information on the spatial and temporal patterns of their release, three relatively new classes of technologies take the center stage, and they share the same molecular detector—GPCRs [114]. There are several approaches that have been developed to detect neuropeptides via high affinity for their ligands [114, 115]. These approaches use different techniques, including genetically encoded reporter systems with the overexpression of chosen components of cellular signaling systems and are based mainly on fluorescence or luminescence [115]. In insects, cell reporter assays are currently commonly used to study ligand‒receptor interactions and the effectiveness of receptor activation not only by native neuropeptides but also by modified molecules) [116]. One of the oldest examples is the discovery of the CNM-amide group of neuropeptides and their receptors, which was performed in 2014 [117].

### Integration with current methods of pest control

Since one of the key aspects of environmental protection is reducing the amount of currently used synthetic insecticides, we are pointing integration of neuropeptide-based strategies with traditional insecticides as a proposition worth considering. To our knowledge, the combined actions of neuropeptides or agents that modulate their pathways and insecticides have not yet been tested. However, this solution seems to be a reasonable way to develop new IPM. Combining both approaches could increase pest control effectiveness while minimizing harm to nontarget species such as pollinators and natural predators. Neuropeptides are less likely to induce resistance, making them a sustainable option. These compounds can also complement biological control methods, such as the use of Cry toxins from *Bacillus thuringiensis*. These substances interfere with signaling pathways, causing, e.g., changes in feeding patterns and causing insect death. It would be worth considering that enriching the activity of cryotoxins with RNAi activity directed towards neuropeptides (or their peptidomimetics), such as AKH [118], could result in disruption of the pro-phenyloxidase cascade; TRPs, which would reduce phagocytic activity, the expression of genes encoding AMPs, and affect food intake and life span [119, 120]. However, our hypothetical approaches need to be confirmed in future studies.

### Policy and regulatory

One of the key challenges associated with innovative pest control methods, such as RNAi or neuropeptides (NPs), is the lack of appropriate legal frameworks enabling their widespread implementation. Examples of the deployment of biological plant protection agents, such as SPEAR^®^ LEP, demonstrate that innovative peptide solutions can effectively replace synthetic insecticides. However, SPEAR® LEP is based on animal-derived neurotoxins rather than neuropeptides. Its effectiveness and selectivity confirm the potential of biotechnology in crop protection but also highlight the need for a clear distinction between different classes of biological compounds.

The implementation of RNAi technologies, neuropeptides, receptor modulators, and gene-editing tools requires strict adherence to international regulations and environmental standards. Differences between the European Union and the United States in terms of insecticide usage guidelines stem mainly from varying regulatory approaches and levels of risk assessment stringency. The EU applies the precautionary principle, meaning that comprehensive studies must be conducted to confirm a substance’s safety for human health, nontarget organisms, and the environment before it can be approved. Under Regulation EC 1107/2009, every active substance must undergo a detailed evaluation by the European Food Safety Authority (EFSA). Moreover, in Europe, pesticide policy is increasingly focused on sustainability and biodiversity protection, as reflected in the European Green Deal. A key component of this strategy is the “Farm to Fork” initiative, which aims to transform the EU’s food system into a more sustainable one. The strategy includes, among other things, reducing the use of chemical pesticides by 50% by 2030 and increasing food safety while minimizing the environmental impact of agriculture (https://www.consilium.europa.eu/pl/policies/from-farm-to-fork/). In the United States, regulations are more flexible and based on risk assessment, meaning that insecticides can be approved more quickly if the available data do not indicate significant threats. The U.S. Environmental Protection Agency (EPA) and the U.S. Department of Agriculture (USDA) assess new substances on the basis of modelling of potential effects and existing scientific studies.

Meeting strict regulatory standards and developing tailored risk assessment frameworks for RNAi, neuropeptides, and receptor modulators are critical steps toward their commercialization. In addition, public acceptance is an important factor influencing the effectiveness and implementation of RNAi technologies. Transparent communication about the benefits and potential risks associated with this technology is essential for its widespread adoption and large-scale legalization.

## Summary

Considering the data presented above, we can conclude that the question raised in the introduction remains truly open: Are we closer to the practical use of neuropeptide-based insecticides or rather molecular techniques targeting neuropeptide-related genes, in pest control? While recent advances suggest a cautiously optimistic 'yes,' the current body of knowledge indicates that widespread implementation is still several years away. This is associated with many issues, including biostability, delivery methods and high risk of action on nontarget animals, including vertebrates. Encouragingly, scientists are aware of all the mentioned challenges and limitations associated with this issue and are constantly striving to solve them. For example, this is expressed in continuous work on the development of biostable peptidomimetics that are successfully patented. However, the methods of peptidomimetic delivery and their specificity still pose significant limitations. To put these perspectives into context, it is worth noting that several peptide-based approaches have already demonstrated promise. For example, combinations of neuropeptide analogues, including CAPA and AKH, were shown to significantly decrease survival and reproduction of the peach–potato aphid (*Myzus persicae*) [121]. These results suggest that neuropeptide-based pest control is moving beyond a purely theoretical concept and may represent an emerging practical approach. Nevertheless, future work should focus on solving two key problems—delivery and specificity. Despite these challenges, and concerns over the cost of peptidomimetic synthesis, limited specificity, and uncertain long-term effects, the field may be turning the corner, with companies such as SOLASTA^®^ Bio aiming to introduce neuropeptide-based targeted insecticides by 2028 (https://solastabio.com/). A potential solution for specificity could be research on the exploring biodiversity of neuropeptides, with perfect examples being natalisins or kinins, and working on different formulations of peptidomimetics or their conjunction with nanoparticles.

As an alternative, current trends in pest control suggest that molecular techniques targeting genes associated with neuropeptides may offer a more realistic and precise solution. Currently used, the previously mentioned market-available SmartStax^®^ PRO maize may point us in the direction of future research on neuropeptides in pest control. Our efforts should be focused on transgenic plants and RNAi technology, which guarantee high specificity of pest control. The first steps have already been taken, among others, by Yue et al. [61, 62]. Our broad knowledge concerning the regulation of crucial physiological processes by neuropeptides will be helpful in the appropriate selection of neuropeptides for knockdown, which significantly affects feeding behavior, development, or pest reproduction. Additionally, a promising prospect could be the integration of methods for knocking down neuropeptide-related genes with currently used methods of pest management, for example, with Cry toxins. However, the introduction of these kinds of solutions to the market requires regulatory and legislative changes. Ongoing regulatory frameworks are insufficiently equipped to address new biotechnological solutions that could be used in IPM. In this situation, researchers and government agencies should cooperate to update current regulations associated with the introduction of new peptide-based insecticides and new strategies based on nucleic acids or gene editing.

## Data Availability

Not applicable.
